# Redox regulation in lifespan determination

**DOI:** 10.1016/j.jbc.2024.105761

**Published:** 2024-02-15

**Authors:** Christina Karagianni, Daphne Bazopoulou

**Affiliations:** Department of Biology, University of Crete, Heraklion, Greece

**Keywords:** ROS, redox signaling, lifespan, aging, stress, longevity pathways

## Abstract

One of the major challenges that remain in the fields of aging and lifespan determination concerns the precise roles that reactive oxygen species (ROS) play in these processes. ROS, including superoxide and hydrogen peroxide, are constantly generated as byproducts of aerobic metabolism, as well as in response to endogenous and exogenous cues. While ROS accumulation and oxidative damage were long considered to constitute some of the main causes of age-associated decline, more recent studies reveal a signaling role in the aging process. In fact, accumulation of ROS, in a spatiotemporal manner, can trigger beneficial cellular responses that promote longevity and healthy aging. In this review, we discuss the importance of timing and compartmentalization of external and internal ROS perturbations in organismal lifespan and the role of redox regulated pathways.

It is now generally accepted that moderate levels of reactive oxygen species (ROS), such as superoxide (O_2_^•−^) and peroxide function to maintain cellular homeostasis and promote biological processes, including growth, metabolism and differentiation (1, 2). While ROS at physiological levels play important roles in cellular signaling, excess of ROS can cause oxidative stress, which is thought to contribute to pathological conditions and ultimately cell death (3). As a result of their ability to cause irreversible oxidative damage to cellular macromolecules, i.e., DNA, proteins, and lipids, ROS were long held responsible for telomere attrition, genomic instability, epigenetic alterations, stem cell exhaustion, cellular senescence, and impaired proteostasis that contribute to aging and age-associated pathologies (4). This hypothesis is known as the “Free Radical Theory of Aging” (FRTA) (5). However, the perspective on ROS and the role that physiological oxidants play in lifespan has shifted dramatically over the past years, primarily due to lack of experimental support for the FRTA. It is now clear that the types of ROS, their relative concentrations as well as their subcellular, and possibly tissue location are all critical factors that ultimately determine whether ROS have beneficial or harmful effects in organisms (6, 7).

Despite the wealth of information on the underlying mechanisms that have emerged in the past 2 decades, the process of aging remains highly complex and establishing cause–effect relationships is a major challenge. One important driver in this research field was the discovery that signaling pathways that regulate longevity are largely conserved across invertebrate and vertebrate species (8). Moreover, although some of these pathways can individually dictate lifespan, there are various points of intersection between them. For instance, mitochondria, are intimately linked to distinct pathways and contribute to specific aspects of the aging process through intracellular signaling (9, 10). To improve our understanding, and eventually to apply targeted interventions, we still need to identify crucial players, characterize their impact on aging and age-associated diseases, and determine which pathways are affected and how. In this review, we address how ROS signaling is linked to organismal lifespan. We discuss the properties of ROS with a focus on hydrogen peroxide (H_2_O_2_) and superoxide O_2_^•−^, the primary ROS species contributing to signaling events, and address previous and current theories on the relationship between ROS and longevity. We also review how ROS production connects with known longevity regulatory mechanisms and how a mild elevation in ROS increases stress responses and lifespan.

## ROS generation, biochemical properties, and clearance

ROS are generated from a variety of sources within the cell. The main sites of ROS production are in the mitochondria. The initial ROS that is formed is O_2_^•−^, a product of electron leakage from the respiratory chain complexes I (NADH: ubiquinone oxidoreductase) and III (ubiquinol-cytochrome c oxidoreductase) (11, 12). Upon its production, O_2_^•−^ is released into the mitochondrial matrix and intermembrane space, respectively (13, 14). A large portion of O_2_^•−^ present in the mitochondrial intermembrane space enters the cytosol *via* voltage-dependent anion channels (15). Another source of intracellular O_2_^•−^ is the incomplete oxidation of endogenous or exogenous substrates (*i.e.,* drugs and xenobiotics) by members of the cytochrome P450 monooxygenase family (16), as well as the membrane-bound NADPH oxidases. These oxidases produce O_2_^•−^ in response to hormonal changes, cell signaling, and pathogens (17, 18, 19). The main targets of O_2_^•−^ are iron–sulfur (Fe–S) clusters, which become unstable and release free iron upon oxidation (20), further exacerbating oxidative stress and macromolecular damage. O_2_^•−^ also reacts with nitric oxide (NO) to form another strong oxidant species (stronger than NO), peroxynitrite (ONOO^−^), known to promote oxidation and nitration reactions (21). Yet, most O_2_^•−^ produced under physiological conditions is rapidly converted into H_2_O_2_ and elemental oxygen by superoxide dismutases (SODs), the major O_2_^•−^ antioxidant defense system. These enzymes are present in virtually all eukaryotes and differ primarily in their active site metals; for instance, mitochondrial SOD contains manganese as the catalytic metal (MnSOD) whereas cytoplasmic or extracellular SODs incorporate copper and zinc (Cu/ZnSOD) into their active sites (22). Most cellular H_2_O_2_ is produced by the dismutation of O_2_^•−^ (23). Other sources of H_2_O_2_ include oxidative protein folding processes in the endoplasmic reticulum lumen. Endoplasmic reticulum oxidoreductin 1 produces H_2_O_2_ by using oxygen (O_2_) as an electron acceptor in the process of transferring disulfides to protein disulfide isomerase, which ultimately oxidizes nascent polypeptide substrates (24). H_2_O_2_ is also produced during fatty acid oxidation by acyl-coenzyme A oxidases in peroxisomes and acyl-coenzyme A dehydrogenases in mitochondria (25, 26, 27). In contrast to O_2_^•−^ (t_1/2_: ∼10^−6^ s, migration distance: ∼30 nm), H_2_O_2_ is a relatively stable and highly membrane diffusible oxidant (t_1/2_: ∼10^−3^ s, migration distance >1 μm) (28). Physiological levels of H_2_O_2_ are important for signaling through the reversible oxidation of target proteins (2). Countless examples exist in which H_2_O_2_ directly oxidizes thiols in cysteine residues. Oxidation leads initially to the formation of sulfenic acid, followed by disulfide bond formation either intramolecularly, or with other protein thiols (RS-SR), or with thiol-containing small molecules, including glutathione, free cysteine or CoA (29). Proteins whose cysteines are reactive toward local changes in peroxide concentrations and undergo reversible oxidation processes either directly or by disulfide exchange with peroxiredoxins (PRXs) are considered redox sensitive. In response to cysteine oxidation, most redox regulated proteins show a change in activity, oligomerization, stability, lipidation, and/or subcellular location, which alters their function (7). In addition, H_2_O_2_ oxidatively modifies methionine (30) and tyrosine (31) residues, reacts with loosely bound metals such as the iron centers of metalloenzymes (32), and targets many of the same iron-sulfur clusters that O_2_^•−^ attacks (20). When encountering Fe^2+^ or Cu^2+^, peroxide generates extremely reactive hydroxyl radicals (^•^OH) through Fenton chemistry (33). While ^•^OH has a limited diffusion potential (t_1/2_: ∼10^−9^ s, migration distance: ∼1 nm), its highly indiscriminate reactivity can directly damage most biomolecules in its vicinity (34).

**Figure 1:**
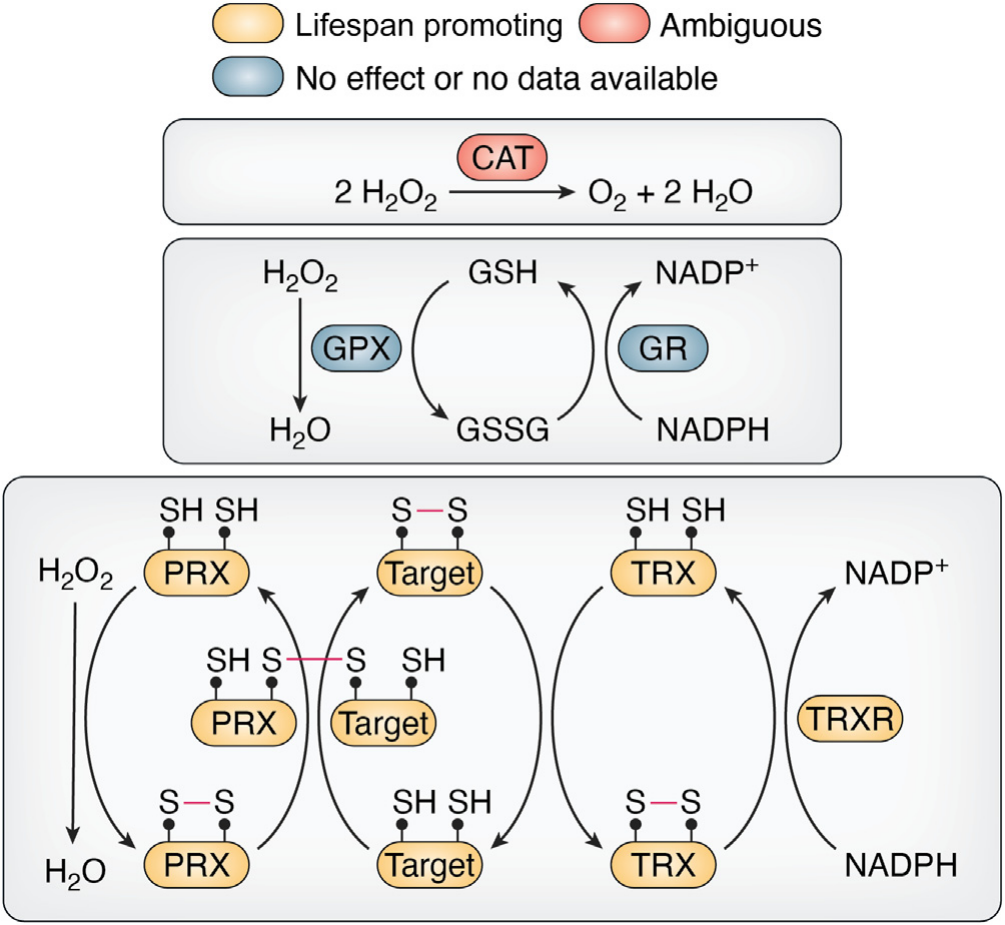
**ROS scavenging and signaling mechanisms.** Shown are contributing redox enzymes with lifespan-promoting (*yellow*), ambiguous (*red*), no or unknown (*blue*) effects on lifespan. CAT, catalase; GPX, glutathione peroxidase; GR, glutathione reductase; GSH, glutathione; GSSG, glutathione disulfide; PRX, peroxiredoxin; TRX, thioredoxin; TRXR, thioredoxin reductase.

Intracellular H_2_O_2_ concentrations are controlled by catalases, PRXs, and glutathione peroxidases (Fig. 1). Whereas catalases catalyze the decomposition of H_2_O_2_ to water and oxygen without the need of reducing cofactors (35, 36), PRXs reduce H_2_O_2_ to H_2_O through a disulfide exchange reaction with thioredoxins (TRXs) (37, 38). Through this interaction with H_2_O_2_, PRXs become rapidly and reversibly oxidized. Their re-reduction ultimately depends on thioredoxin reductase, which uses NADPH as a cofactor. As mentioned earlier, oxidized PRXs couple their catalytic detoxification reaction to a peroxide-mediated signaling role. In this case, the peroxidatic cysteine (C_P_) reacts with H_2_O_2_ to form a transient intermediate, *i.e*., sulfenic acid (-SOH), which condenses with an accessible thiol to form intermolecular (with a target protein) or intramolecular (with the resolving cysteine C_R_) disulfide bonds. The disulfide is then transferred to the target protein *via* thiol-disulfide exchange (39, 40, 41). Glutathione peroxidases catalyze the reduction of cytosolic H_2_O_2_ in the presence of reduced glutathione (GSH), which is subsequently converted into its oxidized state [glutathione disulfide (GSSG)] (42). As the most abundant intracellular thiol source, GSH forms the major redox buffer in most pro- and eukaryotic cells (43). Glutathione reductases reduce GSSG back to GSH using NADPH as an electron donor. As constitutively active enzymes, glutathione reductases function in maintaining a high intracellular GSH:GSSG ratio. This ratio is crucial in determining the redox potential of the cell and serves as an indicator of the level of oxidative stress that cells experience (44).

## Re-evaluating the role of ROS in lifespan regulation

Research spanning several decades suggested that the age-associated increase in ROS levels causes the random oxidation of biomolecules and leads to aging phenotypes and the development of age-associated pathologies (45, 46). Theories that supported this causative role of ROS in the aging process include the FRTA and, a refined version, the “Mitochondrial Theory of Aging’’ (47). According to the Mitochondrial Theory of Aging, ROS produced in the mitochondria cause the accumulation of mutations in mitochondrial DNA (mtDNA), which is particularly vulnerable due to the lack of histone packaging and mitochondrial repair mechanisms (48). Since most mtDNA genes encode for proteins of the electron transport chain (49, 50), mutations in mtDNA typically impair the function of the respiratory chain and ATP synthesis, leading to accelerated ROS production and amplification of the macromolecular damage (47, 51). Initially, many studies appeared to support this hypothesis (Table 1). For instance, most aging model systems, including *Saccharomyces cerevisiae* (52), *Drosophila melanogaster* (53), and *Caenorhabditis elegans* (54) exhibit some amount of age-associated ROS accumulation. Moreover, it was shown that the degree of oxidative damage in macromolecules, particularly proteins (55, 56, 57, 58), correlates with increasing ROS levels, declining GSH:GSSG ratios and increasing age (59, 60, 61). However, follow-up genetic studies were significantly less consistent and painted a much more granular picture. For instance, in *D. melanogaster*, ectopic expression of catalase in mitochondria or overexpression of cytosolic catalase (62, 63) Sod1 (Cu/ZnSOD) or mitochondrial Sod2 (MnSOD) had either no effect (64, 65) or slightly extended lifespan (66, 67, 68). Deletion of *Sod2*, however, resulted in significant lifespan reduction, locomotory dysfunction, and mitochondrial degeneration (69, 70) without increasing the levels of irreversible/toxic protein oxidation (71). In *C. elegans*, the effects of genetic interventions on lifespan seem to depend on the spatiotemporal patterns of the transgene expression and the degree of overexpression. While lack of the main peroxisomal catalase (CTL-2) decreased *C. elegans* lifespan (72), as would be expected from the FRTA, total loss of all SOD activity (SOD-1, -2, -3, -4, and -5) did not (73). On the other hand, overexpression of *sod-1* or *sod-2* extended *C. elegans* lifespan by 20 to 25% (74, 75). In mice, overexpression of SOD1 separately or in combination with catalase or SOD2 was found insufficient to extend lifespan (76, 77). Reduction in SOD2 levels increased the levels of oxidative damage and cancer incidence but did not shorten lifespan (78). Lastly, naked-mole rats, the longest-living rodent, exhibit lower GSH:GSSG ratios and higher levels of oxidative damage compared to shorter-living mice (79).

**Table 1:** Interventions that modulate ROS levels and their effects on lifespan

Function/Target	Change in protein levels/function	Organism	Effect on redox network	Effect on lifespan	Refs
Glutathione reductase	Loss of Glr1 under respiratory conditions	Yeast	Increase in ROS	No effect (replicative lifespan)	(161)
Catalase	Loss of peroxisomal CTL-2	Worm	Increase in protein carbonyls	Decrease in mean lifespan	(72)
	Overexpression of cytosolic Ctl	Fly	No effect in GSH	No effect	(62)
	Ectopic expression of cytosolic Ctl in mitochondria	Fly	No effect in H_2_O_2_	No effect	(63)
	Deletion of catalases	Yeast	Increase in H_2_O_2_	Increase (chronological lifespan)	(111)
	Mitochondrial overexpression of human CTL	Mouse	Decrease in H_2_O_2_	Increase in mean and maximum lifespan	(115)
Superoxide dismutase	Overexpression of Cu/ZnSOD (Sod1) or MnSOD (Sod2)	Fly	No effect	No effect or increase (overexpression in adult flies only)	(64, 65, 66, 67)
	Overexpression of human SOD1 in motor neurons	Fly	No report	Increase in mean and maximum lifespan	(68)
	Loss of Sod2 (globally or in muscles only)	Fly	Increase in O_2_^•−^	Decrease in mean lifespan	(69, 70)
	Loss of SODs (SOD-1 to 5)	Worm	Increase in O_2_^•−^, no effect in protein carbonyls	No effect	(73)
	Overexpression of SOD-1	Worm	Increase in H_2_O_2_ & protein carbonyls	Increase in mean lifespan	(74, 75)
	Addition of SOD mimetics	Worm	No report	No effect	(162)
	Reduced MnSOD activity	Mouse	Increase in DNA oxidation	No effect	(78)
	Overexpression of MnSOD or Cu/ZnSOD	Mouse	Decrease in O_2_^•−^ & lipid peroxidation; no effect in H_2_O_2_	No effect	(76, 77)
Peroxiredoxin	Overexpression of Prx5	Fly	No report	Increase in mean lifespan	(81, 82)
	Loss of Prx5 and Prx3	Fly	Decrease in GSH:GSSG, decrease in sulfhydryls	Decrease in mean lifespan	(80)
	Loss of cytosolic PRDX-2	Worm	Increase in oxidation in specific proteins	Decrease in mean and maximum lifespan	(84, 85)
	Increased Tsa1	Yeast	No report	Increase (replicative lifespan)	(83)
Thioredoxin	Loss of TRX-1	Worm	No report	Decrease in mean and maximum lifespan	(86)
	Overexpression of human TRX	Mouse	No report	Increase in mean and maximum lifespan	(88)
Glutaredoxin	Loss of Grx1 or Grx2	Yeast	Increase in ROS	Decrease (chronological lifespan)	(87)
ETC	Loss of CLK-1 (ubiquinone biosynthesis)	Worm	Increase in ROS	Increase in mean lifespan	(91)
	Loss of ISP-1 (complex III) or NUO-6 (complex I)	Worm	Increase in O_2_^•−^	Increase in mean and maximum lifespan	(90, 131)
	Loss of CCO-1 (cytochrome c oxidase)	Worm	No report	Increase in mean lifespan	(130)
	Knockdown of ND75 (complex I) in muscles	Fly	Increase in O_2_^•−^	Increase in mean lifespan	(94)
	Increased complex I activity (allotopic expression of plant NDI1 NADH dehydrogenase)	Fly	Increase in ROS	Increase in mean lifespan	(53)
	Partial loss of MCLK1	Mouse	Increase in H_2_O_2_	Increase	(98, 99)
	Loss of SURF1 (cytochrome c oxidase)	Mouse	No report	Increase in median lifespan	(100)
NADPH oxidase	Pyrroloquinoline quinone treatment or loss of MEMO-1 or overexpression of BLI-3/NADPH	Worm	Increase in ROS/Activation of peroxidase MLT-7	Increase in mean lifespan	(19, 147)
Insulin/IGF signaling (IIS)	Loss of CHICO (in females)	Fly	Increase in H_2_O_2_	Increase in median lifespan	(128)
	Acute impairment	Worm	Increase in H_2_O_2_	Increase in mean and maximum lifespan	(134)
TOR (target of rapamycin) impairment	Loss of TORC1 or rapamycin treatment	Yeast	Increase in ROS	Increase (chronological lifespan)	(142)
Glutamate-cysteine ligase	Overexpression of GCL in CNS (central nervous system)	Fly	Increase in GSH content	Increase in mean and maximum lifespan	(112)
Transcription factors	Loss of HLH-2	Worm	Increase in H_2_O_2_	Increase in mean lifespan	(148)

Exogenous manipulation	Condition/Compound	Organism	Effect on redox network	Effect on lifespan	Refs
Antioxidants	Vitamin C (ascorbic acid), N-acetylcysteine, α-tocophenol glutathione	Worm, fly, mouse	Decrease in ROS	Various (concentration and/or life-stage dependent)	(104, 107, 108, 109)
Pro-oxidants	Superoxide generators (paraquat, juglone)	Worm	Increase in ROS	Increase	(93, 104, 106)
	*Tert*-butyl hydroperoxide (tBH) during development	Fly	Increase in ROS	Increase in median and maximum	(153)
Dietary restriction (DR)	Glucose restriction	Yeast	Increase in H_2_O_2_	Increase (chronological lifespan)	(111)
	Glucose restriction	Worm	Increase in ROS	Increase	(139)
	Loss of EAT-2 or nutrient-based restriction	Worm	TRX-1 activation in ASJ neurons	Increase in mean lifespan	(137)
Metformin	50 mM	Worm	Increase in H_2_O_2_/Activation of PRDX-2	Increase	(145)
Germline loss	Germline ablation or loss of GLP-1	Worm	Increase in ROS	Increase in mean and maximum lifespan	(146)

ETC, electron transport chain; GCL, glutamate-cysteine ligase.

In contrast to catalases and SODs, whose overexpression or deletion often yields unexpected and contradicting results, genetic manipulation of PRXs or TRXs appears to have more consistent effects on the lifespan of model organisms. *Drosophila* expresses two PRXs, the mitochondrial-specific Prx3 and Prx5 which is additionally found in the cytosol and nucleus. A combined *Prx3*/*Prx5* knockdown decreased the GSH:GSSG ratio and reduced lifespan (80). Expression of *Prx5* in mitochondria, but not in the nucleus or cytosol, conferred a significant rescue effect on longevity, while global expression of *Prx5* was still required for complete restoration of lifespan (81, 82). The early mortality in flies underexpressing both PRXs was also reversed upon the overexpression of TRX reductase whose activity in the reaction coupled to TRX leads to the re-reduction of GSSG (80). An increase in Tsa1, the major PRX in yeast, extended replicative lifespan through the redox-mediated recruitment of chaperones dealing with damaged proteins/aggregates formed during aging or H_2_O_2_ exposure and not through H_2_O_2_ scavenging (83). Moreover, worms depleted of their cytosolic PRX, PRDX-2, exhibited phenotypes that mimic chronic exposure to oxidative stress including reduced lifespan (84, 85). Deletion of the cytosolic TRX, TRX-1, increased oxidative stress sensitivity and shortened lifespan (86). Similarly, deletion of glutaredoxins (GRXs) which, like TRXs, help with reducing protein disulfide bonds and are in turn reduced by glutathione, shortened lifespan (87). Finally, overexpression of TRX increased resistance to oxidative stress and extended lifespan in mice (88). As opposed to catalase and SODs whose sole activity is to reduce H_2_O_2_, PRX-TRX systems are also important for the oxidation-reduction of functional thiol groups and GSSG recycling. The consistent effects of disruptions in the PRX-TRX systems suggest that thiol homeostasis and not H_2_O_2_ scavenging is the critical factor for lifespan determination.

## ROS are beneficial players in organismal lifespan

Mitochondrial function is tightly linked to the aging process in a number of ways (89). Several mutations which impair mitochondrial function such as in *clk-1*, required for ubiquinone biosynthesis, mitochondrial complex III (*isp-1*) or mitochondrial complex I (*nuo-6*) extend *C. elegans* lifespan (90, 91). Moreover, depletion of subunits of the electron transport chain, *e.g., cco-1* (cytochrome c oxidase-1 subunit Vb/COX4), only during *C. elegans* development, was sufficient to increase lifespan (92). These results provided evidence, for the first time, for the temporal dynamics of mitochondrial activity and their effects in lifespan. Subsequent studies demonstrated that the long-lived *isp-1* and *nuo-6* mutants had elevated ROS levels and that O_2_^•−^ was necessary and sufficient for the positive effect on lifespan (93). In flies, a mild knockdown of electron transport chain (ETC) complex I in muscle cells was found to increase ROS levels and prolong lifespan (94). Overall, results from model organisms suggest that ROS produced by mitochondrial functions are important for lifespan extension. Confirmatory studies in mammals are largely missing given that most mutations in ETC components result in developmental lethality or significantly shortened lifespan (95, 96, 97). However, two separate studies reported an increase in lifespan due to a partial loss of mitochondrial function but with a lack of consensus on the implication of mtROS in the process. On the one hand, mice with heterozygous mutation in *Mclk1*, the ortholog of the *C. elegans clk-1*, have decreased ETC capacity, increased production of mtROS, and increased lifespan (98, 99). On the other hand, a knockout of *Surf1*, an assembly factor of complex IV (cytochrome c oxidase), also caused an increase in lifespan accompanied by a mild decrease in mitochondrial respiration but with no change in mtROS production (100, 101). Despite the many studies that support a positive role of mtROS in longevity, knowledge on the mtROS–mediated signaling events and adaptive response processes that are implicated is still limited (see also chapter “ROS targets in lifespan determination”).

Pro-oxidant or antioxidant compounds that exogenously alter ROS levels may also impact lifespan but not in a predictable, unidirectional way. Both compound types can either reduce or extend lifespan depending on their concentration, application time (102, 103, 104), method of administration (105, 106), genetic background (93), and affected ROS species. Vitamin C (ascorbic acid), N-acetylcysteine, α-tocophenol and glutathione shown to neutralize ROS had little or no beneficial effect on longevity at low concentrations but reduced lifespan when administered in high doses or for prolonged times (104, 107, 108, 109, 110). Moreover, two O_2_^•−^ generators, paraquat and juglone, were deleterious in high doses but caused a significant increase in lifespan at low concentrations (93, 104).

## ROS specificity in lifespan determination

Although often used interchangeably as ROS, superoxide, peroxide, hydroxyl radicals etc. have distinct properties based on their intrinsic reactivity, half-life, intracellular source, and local concentration (7). Likely, one of the main reasons for the controversial role of ROS in lifespan determination so far was the fact that ROS were studied as a single entity, generated continuously and ubiquitously. Yet, an increasing number of studies suggest that we need to use a more rigorous approach in studying their effects (Table 1). One example is found in yeast, where the inactivation of catalases or growth under caloric restriction conditions increased H_2_O_2_ levels (and oxidative damage), which in turn reduced O_2_^•−^ levels through the activation of SODs (111). Both conditions promoted yeast’s chronological lifespan. In worms, an increase in O_2_^•−^ specifically protected against oxidative stress and lengthened the lifespan of *isp-1* and *nuo-6* mutants (93).

ROS can have varying effects on lifespan depending on their location. In flies, glutamate-cysteine ligase elevated GSH levels and protected against oxidative stress to a greater extent when overexpressed in the central nervous system rather than globally (112). In worms, an increase in O_2_^•−^ in mitochondria and not in the cytosol, extended the lifespan of *clk-1* mutants (113). Moreover, mitochondrial ROS levels regulated by SOD-3 and PRDX-3 activated translocation of transcription factor KLF-1 from the cytosol to the nucleus *via* p38 MAPK signaling to promote longevity (114).

In mice, targeted overexpression of human catalase in mitochondria protected against H_2_O_2_ toxicity, reduced oxidative DNA damage, and increased lifespan (115). A study aimed to further resolve the site-specificity of ROS showed that ROS produced at different mitochondrial sites (complex I or complex III) caused protein oxidation in distinct sub compartments (116). Moreover, increasing complex I ROS production, specifically from reduced ubiquinone and *via* the reverse electron transport, protected mitochondrial function from oxidative stress and extended lifespan in flies (53).

**Figure 2:**
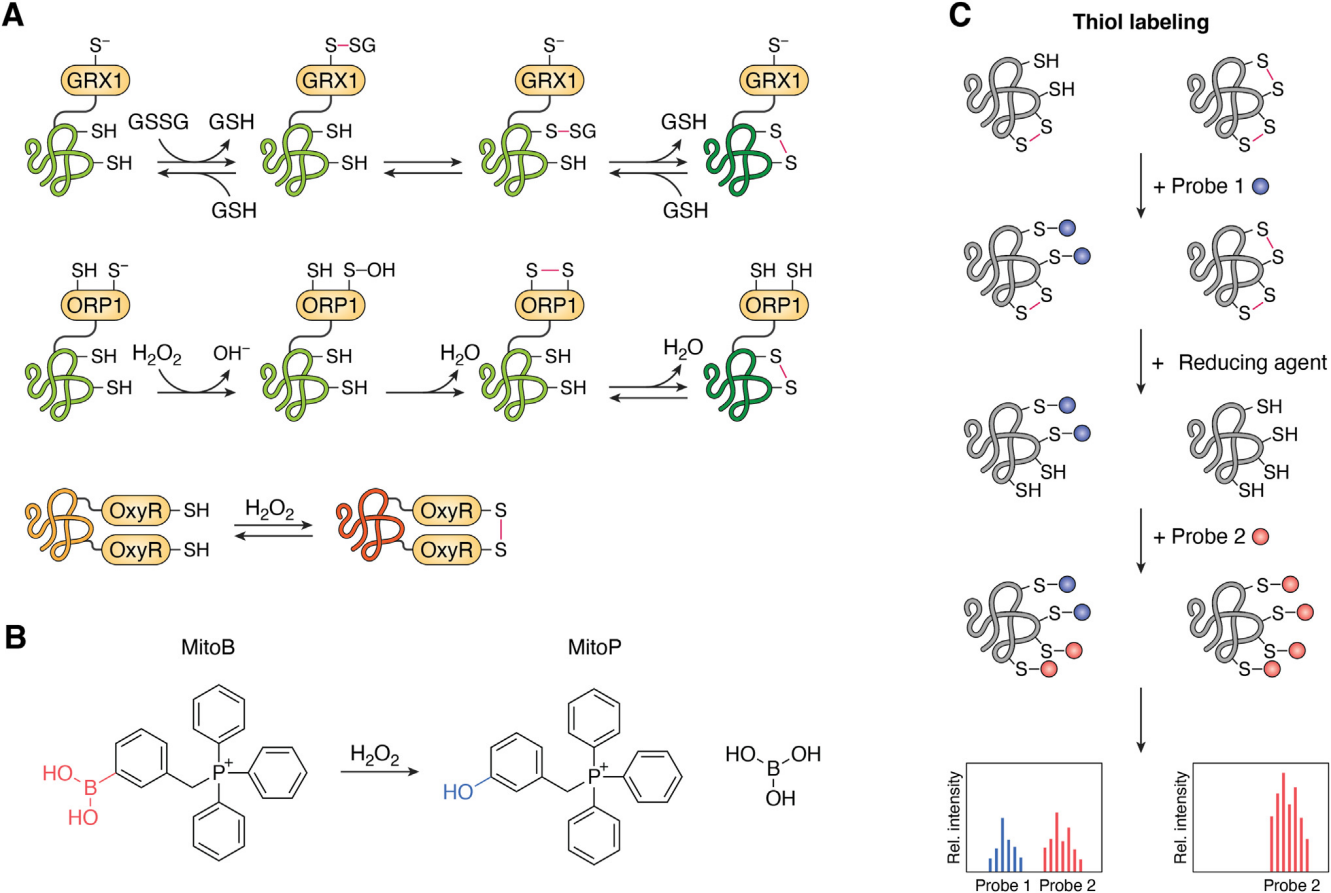
**Measurement of ROS and oxidation *in vivo*.** Redox sensors report on the redox state of their reactive cysteines, depending on the endogenous GSH:GSSG redox couple or their reactivity against specific oxidants (*A*, *B*) (118, 119, 120, 121, 122, 126, 127, 129). Redox proteomics rely on alkyne probes that selectively label cysteine thiols. These probes can be used to globally profile changes in cysteine reactivity due to disulfide formation, which inhibits probe labeling. For example, peptides from different samples can be labeled with isotopically tagged probes (probe 1 or probe 2) and subjected to mass spec analysis to calculate the relative oxidation of individual cysteines within the proteome (*C*) (150). GSH, glutathione; GSSG, glutathione disulfide; GRX1, glutaredoxin; ORP1, oxidant receptor peroxidase 1; OxyR, oxidative stress regulator.

*In vivo* probes that are either selective for specific ROS or report on the activity of endogenous thiol reactive redox systems, such as TRXs and GRXs, have significantly increased the resolution and sensitivity in measuring oxidative stress events (117) (Fig. 2, *A* and *B*). Genetically encoded reduction–oxidation sensitive green fluorescent protein (roGFP) probes alter the redox state of their engineered reactive cysteines and equilibrate with the glutathione redox couple (GSH:GSSG) in a reaction catalyzed by endogenous GRXs. Fusion of roGFP2 to the mammalian Grx1 circumvents dependency of the measurements on the availability of endogenous GRXs (118). Fusion of a thiol peroxidase domain to roGFP mediates high specificity to H_2_O_2_. One such example is the fusion of roGFP2 with the yeast oxidant receptor peroxidase-1, which is employed as a H_2_O_2_-sensitive probe in worms, flies, plants, and mammalian cells (119, 120, 121, 122). Other roGFP2-PRX fusions with selective reactivity toward H_2_O_2_ are the roGFP2-Tsa2ΔC_R_ (123), roGFP2-Tpx1 (124), and roGFP2-PRX2 (125) probes. HyPer-based sensors are another type of H_2_O_2_-sensing probes widely used in redox imaging which, instead of PRXs, employ the redox-sensitive bacterial transcription factor oxidative stress regulator (126, 127). Studies in flies using probes reporting on the GSH:GSSG redox couple and H_2_O_2_ confirmed that age-dependent, pro-oxidative changes exist but are oxidant specific and highly restricted to tissues and compartments (128). In flies, quantifications using the mass spec probe MitoB, which is sensitive to H_2_O_2_ but may also respond to ONOO^–^, confirmed an increase in H_2_O_2_ levels with age (129).

## ROS targets in lifespan determination

Presently, there is solid evidence regarding the positive effects of ROS in lifespan through the function of repair and survival mechanisms (Table 1). Inactivation of ETC components can prolong lifespan by distinct mechanisms involving ROS (90). One such mechanism is triggered by knocking down *cco-1* and implicates the mtROS-induced mitochondrial unfolded protein response (130). Mutations in ETC subunits that cause elevated mtROS (*i.e., isp-1*, and *nuo-*6) can also engage the intrinsic apoptotic pathway and activate a protective response which extends lifespan instead of promoting cell death (131). Mutations in *clk-1* and *isp-1* have also been shown to activate hypoxia-inducible factor 1 *via* elevated ROS levels to stimulate gene expression and extend longevity (132). Lactate and pyruvate cause mild ROS elevations which increase *C. elegans* lifespan and stress resistance *via* the unfolded protein response in the ER and p38 MAPK pathways (133).

**Figure 3:**
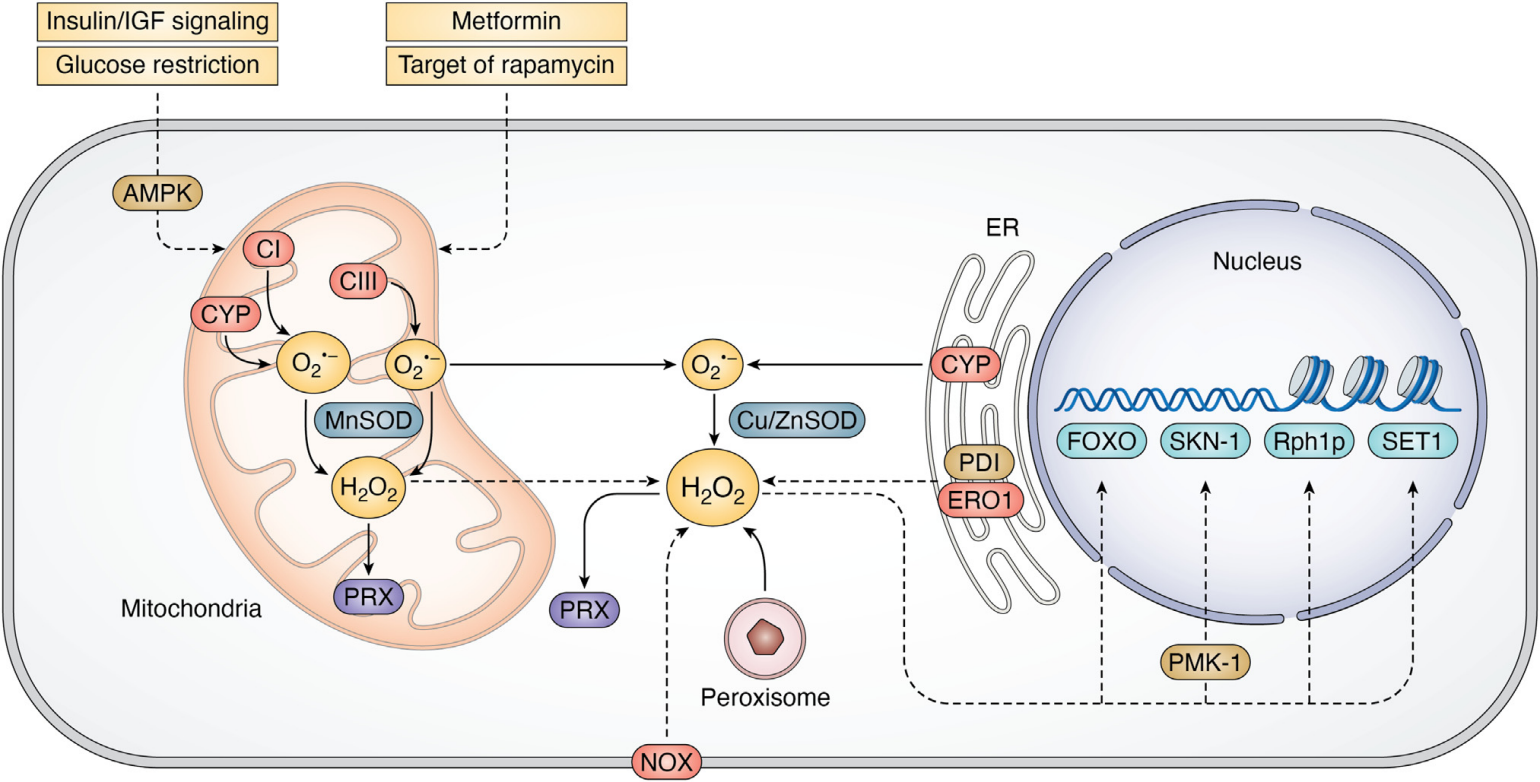
**Key modulators and targets of ROS that impact lifespan.** The *solid lines* indicate known and direct interaction, transition, or ROS production; the *broken arrows* indicate mechanisms requiring further investigation. Shown are ROS sources (*red*) and scavengers (*blue*), redox relays (*purple*), redox-sensitive targets (*green*), and other associated proteins (*orange*). AMPK, AMP-activated protein kinase; CI, complex I in mitochondrial electron transport chain; CIII, complex III in mitochondrial electron transport chain; CYP, cytochrome P450 monooxygenase; ERO1, ER oxidoreductin 1; FOXO, forkhead box transcription factor; NOX, NADPH oxidase; PDI, protein disulfide isomerase; PMK-1, p38 mitogen-activated protein kinase; PRX, peroxiredoxin; Rph1p, H3K36 demethylase (yeast); SET1, H3K4 methyltransferase; SKN-1, *C. elegans* functional ortholog of the mammalian Nrf2 transcription factor; SOD, superoxide dismutase.

Evidence of ROS implication in known longevity-regulating mechanisms is constantly growing (Fig. 3). An impairment in the insulin/IGF signaling (IIS) pathway, a well-studied stress-sensing and lifespan determining pathway, increases lifespan through the activation of transcription factor DAF-16/FOXO and is accompanied by an increase in H_2_O_2_ (134). Elevated ROS triggered by long-lived mitochondrial mutations (*i.e., clk-1*, *nuo-6*, and *isp-1*) also cause activation of DAF-16, indicating that different longevity mechanisms converge (135).

Dietary restriction (DR), the most universal anti-aging intervention, although protective from oxidative damage, has little effect on ROS production or detoxification (129, 136). However, evidence of ROS signaling as an integral part of DR mechanisms is mounting. TRX-1 is necessary for the lifespan extension in *C. elegans* under genetically induced DR (*eat-2* mutation) (137). Glucose restriction, caused by either a chemical inhibitor or by acutely impaired IIS, increases mitochondrial respiration, activates the energy sensor AAK-2/AMPK (AMP-activated protein kinase) (138), and generates mtROS (139). This mtROS signal engages the stress-activated transcriptional factor SKN-1 (Nrf2) *via* the p38 MAP kinase PMK-1 to promote stress resistance and lifespan. Inactivation of target of rapamycin), a nutrient-sensor implicated in the DR pathway, causes lifespan extension in a wide range of organisms (140, 141) and is linked to elevated mtROS production (142). In yeast, the PRX Tsa1 is required for the increase in H_2_O_2_ resistance and lifespan upon caloric restriction, by redox-regulating protein kinase A (143).

Metformin, an antiglycemic drug that targets several pro-aging pathways (144), increases H_2_O_2_ levels and extends lifespan *via* PRDX-2 (145). ROS levels are also increased in *C. elegans* germline-deficient mutants and appear to contribute to the mechanism by which germline loss extends lifespan (146). Fine-tuned production of H_2_O_2_ by the BLI-3/NADPH oxidase can increase longevity through the PMK-1/SKN-1 pathway (19, 147). Impairment of HLH-2/Tcf3/E2A, a conserved transcription factor, also extends *C. elegans* lifespan *via* H_2_O_2_-mediated signaling and regulation of known longevity pathways (AAK-2/AMPK, LET-363/mTOR, SKN-1, and HSF-1) (148). Besides transcription factors, ROS signals can also affect gene expression by targeting epigenetic modifiers. ROS regulation of histone demethylase Rph1p causes a reduction of H3K36 trimethylation and leads to transcriptional silencing which extends yeast’s chronological lifespan (149).

Chemical-proteomic approaches such as cysteine-reactivity profiling (also known as thiol-redox proteomics) (150) can serve to identify cysteine oxidation events in proteins directly targeted by ROS. These approaches rely on alkylating probes which can irreversibly attach to free (reduced) thiols. Due to the reversible nature of many oxidative thiol modifications, these probes enable initially reduced cysteines to be differentially labeled with one probe and reversibly oxidized cysteines to be labeled with another probe after reduction (Fig. 2*C*). Redox proteomics in *C. elegans* confirmed the presence of redox-sensitive target proteins in the p38 MAPK pathway (151) as well as changes in cysteine reactivity due to impaired IIS (152).

Despite having a limited half-life and localized action, ROS can initiate signaling with long term, systemic effects depending not only on the production site and intensity but also on the life stage. During fly development, low doses of oxidants promote longevity *via* “antibiotic-like” depletion of specific bacteria from the microbiome (153). In worms, early life exposure to mitochondrial stressors can elicit positive, life-long effects, attributed to redox-mediated signaling *via* SKN-1 (154). Moreover, developing worms with a stochastically more oxidizing intracellular environment show increased stress resistance and lifespan due to a global reduction of histone H3K4 trimethylation, a known longevity-regulating mechanism (155). This reduction is caused by the reversible, redox-mediated inactivation of the *C. elegans* SET-2, a homolog of the mammalian SET1/MLL H3K4 methyltransferase (156).

## Conclusion and perspective

**Figure 4:**
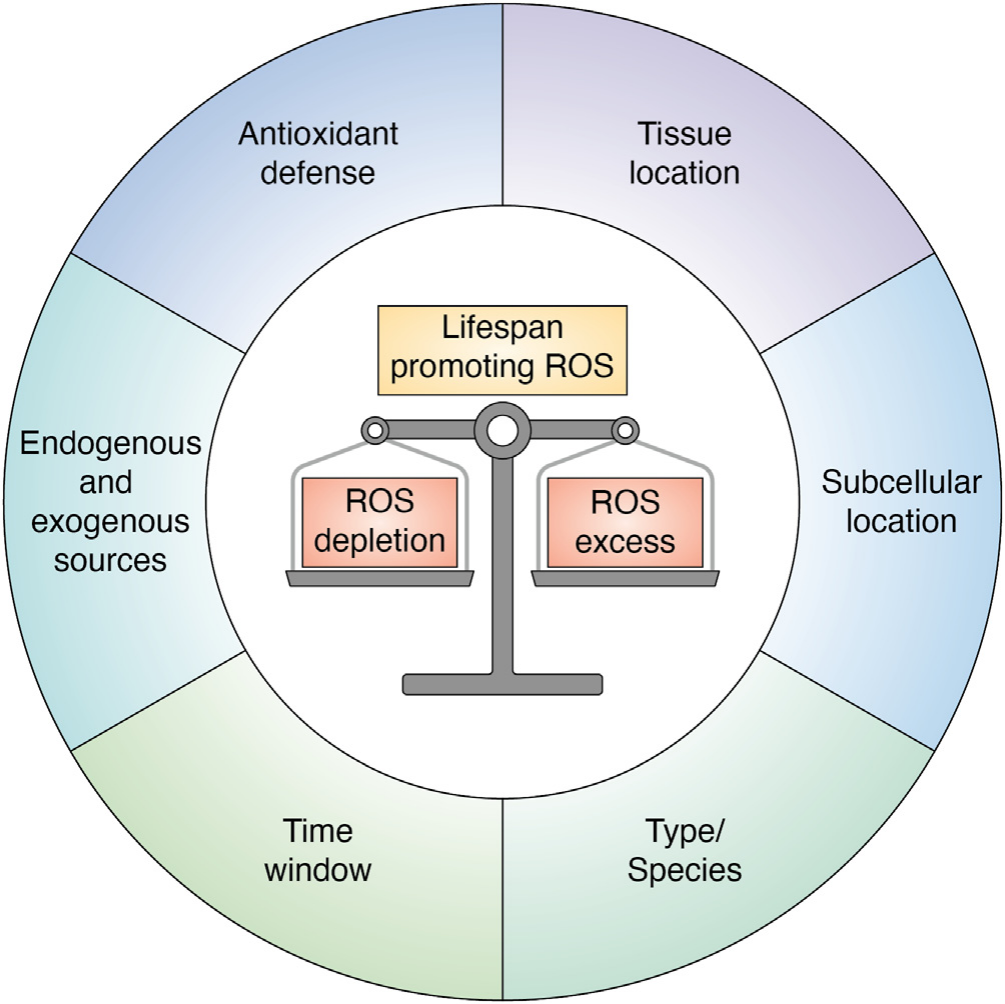
**The complex relationship between ROS and lifespan.** ROS are beneficial as mediators of redox signaling and their moderate production in model organisms can extend lifespan depending on the timing, site, levels, and species. ROS, reactive oxygen species.

It is currently accepted that dysregulation of ROS is linked to the physiological decline that comes with age. However, details on the role of ROS as signaling agents in the aging process are largely lacking. An important step forward would be the identification of specific redox targets *i.e.,* reactive cysteine residues within proteins and the understanding of their relationship to the aging process. Redox proteomics have provided evidence that age is not associated with an increase in nonspecific protein oxidation. Instead, aging correlates with a loss in redox-regulated sites, in a tissue-specific manner (157). Future studies on mapping the redox network across tissues, life stages, and under genetic or pharmacological manipulations will expand our understanding of this age-dependent remodeling and its functional consequences on lifespan (Fig. 4).

Redox biosensors have enabled time-resolved and localized ROS detection (158) and continue to evolve to address the growing diversity of the role ROS play in terms of molecule specificity, concentration, and kinetics. The development of fluorescent redox sensors for multispectral detection will allow combining ROS monitoring with other cellular markers or indicators (*e.g*., pH, ions). Moreover, optogenetic, *i.e.,* light-sensitive ROS-generating proteins (159), and chemogenetic, *i.e*., D-amino acid oxidase tools (160), have recently emerged to provide spatiotemporal control over ROS production. These advances in monitoring and modifying local redox states will be crucial to fully characterize transient redox signals.

The role of ROS has extended far beyond sustaining organismal function. Mild elevations in ROS can lead to adaptation to external perturbations and increase resilience to age-dependent decline. Interventions that prevent oxidant overload and mitigate nonspecific oxidative damage are important in slowing aging. Moreover, more targeted therapeutic and lifestyle-based strategies that fine-tune ROS levels to supply redox reactions and enhance stress resistance and lifespan will be key as we move forward.

## Conflict of interest

The authors declare that they have no conflicts of interest with the contents of this article.
